# Aggregation-Induced Emission-Based Chemiluminescence Systems in Biochemical Analysis and Disease Theranostics

**DOI:** 10.3390/molecules29050983

**Published:** 2024-02-23

**Authors:** Yixin Shi, Xuewen He

**Affiliations:** The Key Lab of Health Chemistry and Molecular Diagnosis of Suzhou, College of Chemistry, Chemical Engineering and Materials Science, Soochow University, Suzhou 215123, China

**Keywords:** chemiluminescence, aggregation-induced emission, molecular sensing, bioimaging, disease therapy

## Abstract

Chemiluminescence (CL) is of great significance in biochemical analysis and imaging due to its high sensitivity and lack of need for external excitation. In this review, we summarized the recent progress of AIE-based CL systems, including their working mechanisms and applications in biochemical analysis, bioimaging, and disease diagnosis and treatment. In ion and molecular detection, CL shows high selectivity and high sensitivity, especially in the detection of dynamic reactive oxygen species (ROS). Further, the integrated NIR-CL single-molecule system and nanostructural CL platform harnessing CL resonance energy transfer (CRET) have remarkable advantages in long-term imaging with superior capability in penetrating deep tissue depth and high signal-to-noise ratio, and are promising in the applications of in vivo imaging and image-guided disease therapy. Finally, we summarized the shortcomings of the existing AIE-CL system and provided our perspective on the possible ways to develop more powerful CL systems in the future. It can be highly expected that these promoted CL systems will play bigger roles in biochemical analysis and disease theranostics.

## 1. Introduction

CL is a kind of photophysical phenomenon in which chemical energy is converted into luminescence in the process of chemical reaction. Because of its high signal-to-noise ratio and high sensitivity, CL is widely used in the development of immune detection kits. At the same time, it has the advantages of in situ tracing and deeper tissue penetration depth in biological imaging as it has no need for external light excitation. Further use of CRET strategies can further red-shift the luminescence signal, thus further reducing the background in the bioimaging, showing a higher signal-to-noise ratio and higher penetration depth. At present, CL-based systems have been employed in ion detection, such as mercury ions [1], cyanide anions [2], and nitrite [3]. They also display excellent performance in ROS detection [4,5,6], molecule detection [7,8,9], bioimaging [10,11,12,13], and disease diagnosis and treatment [14,15,16,17,18]. However, these systems are usually based on very limited CL substrate molecules, suffering weak signal and poor capability in antienvironmental interference.

As a new type of fluorophore, aggregation-induced emission luminogens (AIEgens) with rotator structures possess significantly different characteristics and advantages compared to traditional organic dyes [19,20]. In dilute solutions or dispersed states, AIEgens usually exhibit non-emission or weak emission, whereas in the aggregated state or after binding to the target, the non-radiative transition channel of the excited state is limited by inhibiting the intramolecular motion, leading to a significantly enhanced emission signal. Because of its remarkable advantages such as high quantum yield, tunable emission wavelength, strong photobleaching resistance, large Stokes shift, good biocompatibility, etc., AIE molecules and materials have been widely used in the detection of ion or molecular targets, cellular and subcellular imaging, real-time tracking of dynamic bioprocesses, and image-guided disease therapy [21,22,23,24,25]. Because they can further enhance the intensity and improve the stability of signals, AIE-based CL systems have gradually attracted the interest of researchers. In addition, through molecular engineering, response/targeting groups can be incorporated into the AIE-CL structure, and the emission wavelength can be extended to the near-infrared (NIR) range, opening up a new direction for the sensitive detection of targets and accurate imaging in living systems.

In this review, we mainly focus on the mechanisms and biomedical applications of AIE-based CL systems, such as the molecular design principle, analyte sensing, bioimaging, and disease theranostics. These include (1) direct CL and indirect CL, in which direct CL includes intramolecular electron exchange and intermolecular electron exchange initiated by chemical reactions, and indirect CL includes intramolecular CRET effect and intermolecular CRET effect; (2) detection of heavy metal ions (e.g., mercury ions) and environmentally harmful ions (e.g., nitrite ions) as well as molecular analytes (e.g., peroxide, hydrazine); and (3) CL systems applied in NIR and long-term imaging in living cells and in vivo as well as disease diagnosis and treatment. Through an overview of the recent progress of AIE-based CL systems, we expect to provide some valuable guidance for the subsequent design of new CL systems with longer imaging time, stronger/more stable emission signals, and the capability to perform multi-target sensing simultaneously. It holds great potential for providing more accurate information and more powerful tools and platforms for environmental detection, biochemical analysis, and disease diagnosis and treatment.

## 2. The Working Mechanism of CL Systems

**Figure 1:**
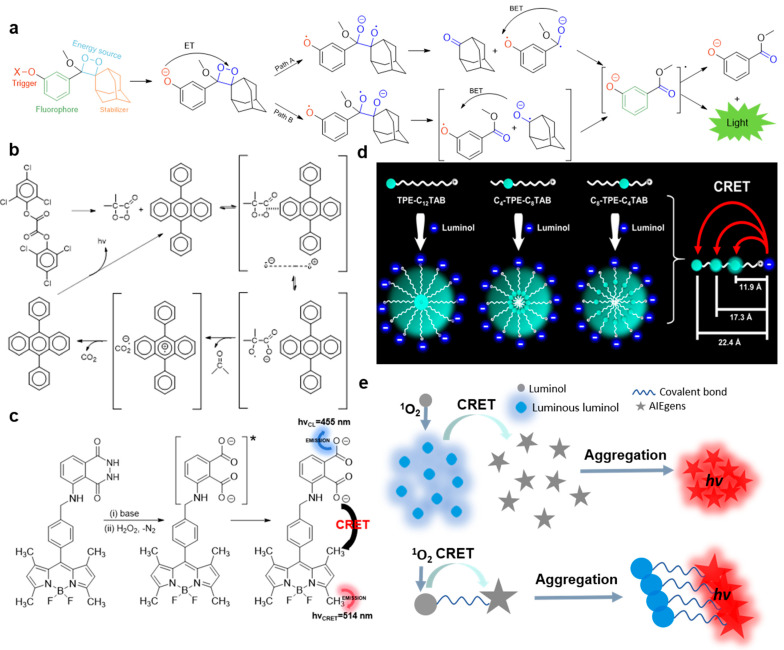
(**a**) The molecular structure and plausible intramolecular CIEEL mechanistic pathway of Schaap’s dioxetane CL emission. (**b**) Intermolecular CIEEL mechanism of TCPO-based CL system. (**a**,**b**) were modified from [26]. (**c**) Corresponding mechanism of BODIPY–luminol CL system with dual emissions, modified from [28]; * signifies excited state. (**d**) Preparation of three kinds of AIE-active cationic micelles for the quantitation of CRET efficiency by precisely tuning the donor–acceptor distance, from [29]. (**e**) Schematic illustration of intramolecular and intermolecular CRET of AIE-CL system.

The CL mechanism of Bis (2,4,6-trichlorophenyl) ethanedioate (TCPO) is intermolecular chemically initiated electron exchange luminescence (CIEEL), as shown in Figure 1a. In the presence of TCPO, hydrogen peroxide and activators (such as rubrene (RUB), perylene (PER), 9,10-diphenylanthracene (DPA), anthracene (ANT), 2,5-diphenyloxazole (PPO), 9,10-dimethoxyanthracene (DMOA), 9,10-dicyanoanthracene (DCNA), etc.), the high-energy intermediate 1,2-dioxadione is first formed, and then the charge transfer occurs between the activator and 1,2-dioxadione. During the elongation and breaking of the O–O bond of the high-energy intermediate, the intermolecular electron transfer from the activator to 1,2-dioxanone takes place irreversibly. Subsequently, the C–C bond is broken, resulting in the formation of new free radical ion pairs in the solvent cage. Back electron transfer populates the chemiexcited singlet state of the activator and finally emits CL [26]. A typical example of the mechanism of intramolecular CIEEL is the CL from dioxetanes. In this system, dioxetanes are the source of energy. With the removal of the Trigger group, the intramolecular electrons are transferred from the phenolate ion to dioxane groups, forming high-energy dioxane, leading to the breaking of the O–O bond and resulting in intramolecular CIEEL. Since the cleavage of the O–O bond can form compounds **2a** or **2b** (Figure 1a), there are two possible ways to induce CL: for path A, intermediate **2a** is decomposed into **3a** and adamantanone, and then intramolecular back electron transfer leads to excited state 4 (4*), which releases CL during its decay to ground state 4. For path B, compound **2b** is decomposed into the solvent cage-like radical ion pair **3b**, followed by back electron transfer to form an excited state 4 (4*), which releases CL during the transition back to ground state 4 [26].

CRET is the non-radiative transfer of energy from CL reagents to energy receptors while chemical reactions take place. As shown in Figure 1e, CRET can occur at the intermolecular or intramolecular level. Tang and Zou reported a kind of AIE-active TPE-SDS which can amplify the intrinsic CL of singlet oxygen (^1^O_2_) [27]. The intrinsic emission of ^1^O_2_ partially overlaps with the absorption peak of TPE-SDS at 366 nm. In the presence of TPE-SDS, a new strong emission peak corresponding to the emission of TPS-SDS is detected in an IO_4_-H_2_O_2_ system. The energy exchange between ^1^O_2_ and TPE-SDS can be regarded as the intermolecular CRET. J. Rochford et al. designed and synthesized a BODIPY–luminol-conjugated compound [28]. In a Na_2_CO_3_/NaHCO_3_ solution with a pH value of 10, luminol can emit blue CL at 455 nm under the catalysis of CuSO_4_/H_2_O_2_, which overlaps with the absorption peak of BODIPY. Double emission of BODIPY–luminol can be observed in H_2_O_2_ and CuSO_4_ solutions. The energy transfer between luminol and BODIPY within the BODIPY–luminol molecule can be regarded as the intramolecular CRET.

One of the critical factors that determines the efficiency of CRET is the spatial distance between the CL agent and the receptor. Three kinds of AIE-anchored cationic surfactants with AIE activity were synthesized by Lu’s group [29]. By forming micelles, the distance of luminol to the nucleic TPE domain was precisely tuned from 11.9 Å to 22.4 Å. The CRET efficiency was therefore quantitatively measured and proved to be inversely proportional to the sixth power of the distance between the luminol donor and TPE receptor, consistent with the Förster resonance theory.

## 3. Application in Biochemical Analysis In Vitro

### 3.1. Ion Detection

As mercury ions (Hg^2+^) are emanated to their surroundings in the course of various natural events and human activities, the accurate sensing of Hg^2+^ is essential for human health and environmental protection [30]. For fast, sensitive, and selective detection of Hg^2+^, Hou’s group developed a new AIE-CL sensor based on the enhancement of the CL signal of a TCPO-H_2_O_2_ system by aggregated-state gold complexes (Au(I)–thiolate complexes with AIE properties) [1]. Due to the strong affinity of thiol groups in Au(I)–thiolate complexes with Hg^2+^, the aggregation of Au(I)–thiolate complexes is disrupted in the presence of Hg^2+^, resulting in the turn-off of the CL signal of the TCPO-H_2_O_2_ system. The detection limit of Hg^2+^ was as low as 3.0 ng·mL^−1^ with a linear detection range of 0.005–10 μg·mL^−1^. The exposure level of Hg^2+^ in water was successfully determined and the contamination distribution of Hg^2+^ can be fast and sensitively surveyed. 

**Figure 2:**
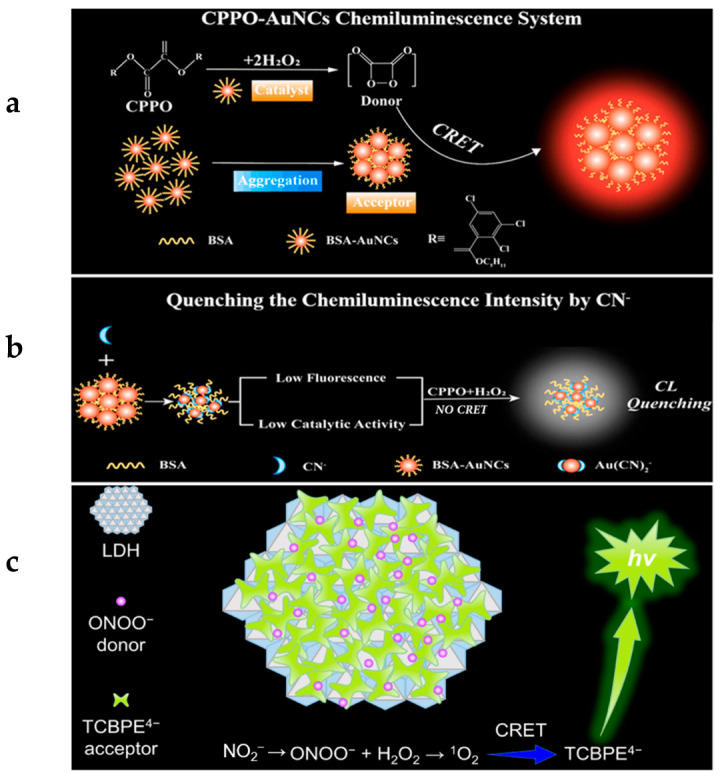
(**a**) Schematic diagram of the CL mechanism of AuNCs-CPPO system. (**b**) Schematic illustration of the CL intensity quenching by CN^−^. (**a**,**b**) are from [2]. (**c**) Schematic diagram of introducing the random assembly of LDH-supported AIE acceptor into the ONOO−CL system for achieving ultrahigh CRET efficiency, modified from [3].

Detection of cyanide ions (CN^−^) is of essential importance due to their high toxicity to human life [31]. To sensitively detect CN^−^ in environmental water samples, Zhang’s group developed a gold nanocluster (AuNC)–peroxyoxalate integrated CL system. The high-energy intermediate was formed by the CL reaction of Bis (2,4,5-trichloro-6-carbopentoxyphenyl) oxalate (CPPO) and hydrogen peroxide (H_2_O_2_) (as shown in Figure 2a,b) [2]. The energy was transferred to the AuNC which was the receptor of the CRET process. In addition, the AuNC can serve as a catalyst to promote the formation of high-energy intermediates and has an AIE effect. In the presence of CN^−^, the CL signal was obviously quenched due to the Elsner complex reaction between CN^−^ and AuNCs. The CN^−^ in environmental water samples was sensitively detected. In total, 16 types of anions (F^−^, Cl^−^, Br^−^, I^−^, CO_3_^2−^, NO_3_^−^, NO_2_^−^, SO_4_^2−^, C_2_O_4_^2−^, CH_3_COO^−^, SCN^−^, PO_4_^3−^, S_2_O_3_^2−^, citrate ions, SO_3_^2−^, and EDTA^2−^) and 4 common cations (K^+^, Na^+^, Ca^2+^, and Mg^2+^) were chosen as controls. Only 100 μg/L CN^−^ caused a sharp decrease in chemiluminescence intensity, showing that its anti-interference ability is much higher than that of the reported sensing technology [32,33,34,35]. In addition, the AuNC-CL system has a linear detection range of 2.5~125 μg·L^−1^ and a detection limit down to 0.55 μg·L^−1^, which is 10 times lower than that of a AuNC sensor with a single fluorescence mode.

As nitrite has a high probability of causing food poisoning and potential carcinogenic effects, its sensitive detection is significantly important in the food industry and for human health [36]. As shown in Figure 2c, Guan and coworkers designed a powerful CL system for the sensitive detection of nitrite by taking advantage of an efficient CRET process using luminol as the donor and negatively charged Na_4_TCBPE with AIE activity as the CRET receptor, which orderly assembled on positively charged layered double hydroxides (LDHs) to shorten the donor–acceptor distance and improve the efficiency of the CRET effect [3]. Additionally, before and after the chemiluminescence reaction, almost no change was observed in the optical signal intensity of LDH-supported Na_4_TCBPE, showing that LDH-supported Na_4_TCBPE has high stability and renewability. In addition, the adsorption of ONOO^−^ can further effectively shorten the distance between the CL donor and the AIE acceptor, thus obtaining efficient CRET. The proposed CL system has been successfully applied to the detection of nitrite in the concentration range of 1.0~100 μM with a detection limit down to 0.5 μM. This excellent stability and reproducibility further verify its great potential in practical applications.

### 3.2. Reactive Oxygen Species (ROS) Detection

ROS is a collective term for the reactive metabolites of oxygen that are prevalent in aerobic organisms, including free radicals (e.g., superoxide anion (O_2_^•−^), hydroxyl (^•^OH), hydroperoxyl (HO_2_^•^), alkyl (RO^•^), and peroxyl (RO^•^)), non-radicals (e.g., hydrogen peroxide (H_2_O_2_), singlet oxygen (^1^O_2_), and excited carbonyl (RO*)), and certain acids (e.g., hypochlorous acid (HOCl), hypoiodous acid (HOI), and hypobromous acids (HOBr)) [37]. ROS are normally formed in organisms as a natural by-product of oxygen metabolism and have an important role in cell signaling and homeostasis in vivo [38]. However, under abnormal stimuli, ROS levels increase dramatically, which may cause serious damage to cellular structures [39]. Therefore, it is of great significance to design AIE-CL systems that can efficiently detect ROS.

**Figure 3:**
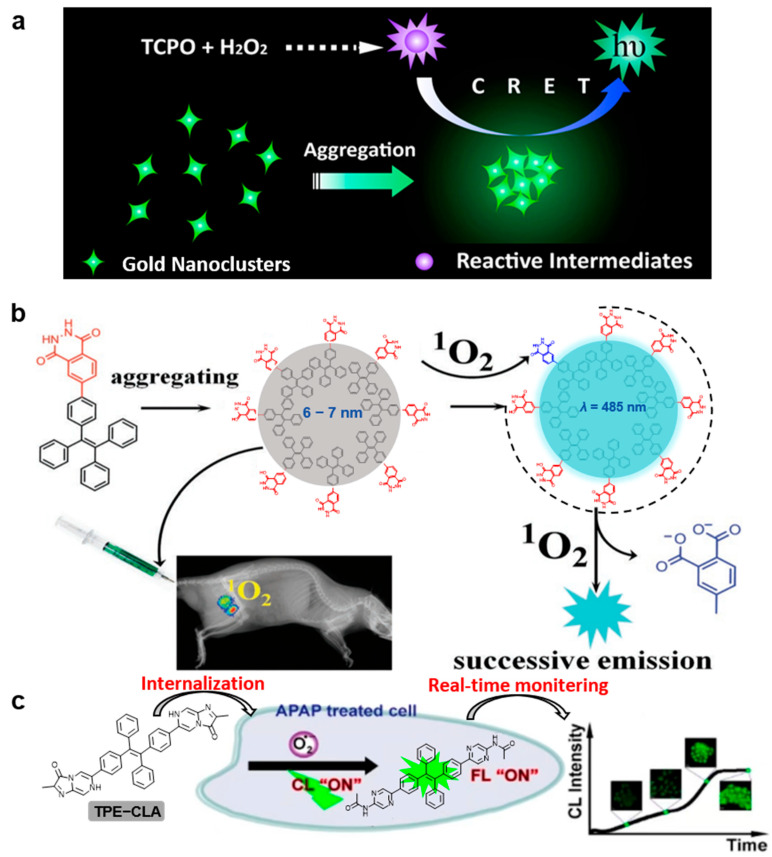
(**a**) Schematic illustration of gold nanocluster aggregate-amplified TCPO–H_2_O_2_ CL, from [4]. (**b**) Schematic representation of the NTPE-PH formation and its CL response to ^1^O_2_, from [5]. (**c**) Chemical structure and proposed turn-on mechanism of the TPE-CLA strategy with FL/CL dual detection signals, modified from [6].

In order to avoid the CL quenching effect caused by aggregation, there is a great demand to design a new generation of CRET acceptors with an AIE character. Lu’s group reported an AIE-active AuNC system, which can significantly amplify the CL signal of a TCPO-H_2_O_2_ system through the CRET effect (shown in Figure 3a) [4]. This strategy can be applied to the detection of H_2_O_2_ with a detection limit of 2.0 µM, lower than that of an imidazole-catalyzed Rhodamine B system.

Because the lifetime of singlet oxygen (^1^O_2_) is as low as microseconds and is easily quenched by a variety of reductants in tissues, the detection of ^1^O_2_ in animals is considered to be one of the most challenging tasks via non-invasive technology. As shown in Figure 3b, Lv’s group reported a powerful CL nanosensor (NTPE-PH) which has an ultrahigh uploading concentration of CL unit in the nanosensor [5]. Attributed to the highly efficient intramolecular energy transfer and the strong AIE activity, the CL signal was remarkably amplified. Compared to superoxide anion radical (O_2_^•−^), H_2_O_2_, hydroxyl radical (OH^•^) and hypochlorite (ClO^−^), NTPE-PH has great specificity toward ^1^O_2_. In addition, experiments show that NTPE-PH has good stability in water. Nine Dark Agouti rats were treated with 1.0 mL of 1.0 × 10^−3^ M NTPE-PH three times a week, and all the rats survived normally after three months, indicating that NTPE-PH has great biocompatibility and low toxicity. The NTPE-PH sensor can selectively and sensitively respond to ^1^O_2_ within a nanometer distance. The linear quantitation of ^1^O_2_ was realized in the range of 10 nM~10 µM, and the detection limit was as low as 4.6 nM.

Superoxide anion (O_2_^•−^), as the precursor of other intracellular ROS, has both protective and harmful effects in the body [37]. Thus, it is of great significance to accurately monitor the dynamic changes of O_2_^•−^. Tang’s group constructed a novel organic probe, denoted as TPA-CLA, with typical AIE properties (Figure 3c) [6]. The solubility of the probes decreased and aggregates formed after interaction with O_2_^•−^, thus turning on the FL and CL signals. The TPE-CLA was highly sensitive to O_2_^•−^, and the detection limits of the FL and CL modes were 0.21 nM and 0.38 nM, respectively. Meanwhile, the specificity of TPECLA to O_2_^•−^ was also explored, which showed that FL/CL signals can be inhibited by SOD (superoxide dismutase, a scavenger of O_2_^•−^) and other reactive species showed negligible FL/CL responses. In addition, the control group showed obvious CL signals after injection of TPE-CLA without a significant change during the whole experimental process, indicating the excellent stability of the TPE-CLA. Further, the imaging of natural O_2_^•−^ in Raw264.7 cells and the stimulated O_2_^•−^ in the inflammatory mice was successfully realized.

### 3.3. Molecular Sensing

Traditional absorption-based molecular sensing methods like the colorimetric approach provide convenience for signal readout, yet they usually suffer from low sensitivity and a short linear range for detection [40,41,42,43]. Although fluorescence signals are much more sensitive (>1000-fold) than absorption modes, the shortcomings of photobleaching and low noise-to-signal ratio impede its wide practical application [44,45,46,47,48,49,50,51,52,53,54,55,56]. Alternatively, CL detection shows great potential in terms of high-sensitivity detection with a high signal-to-background ratio and no need for external light excitation.

**Figure 4:**
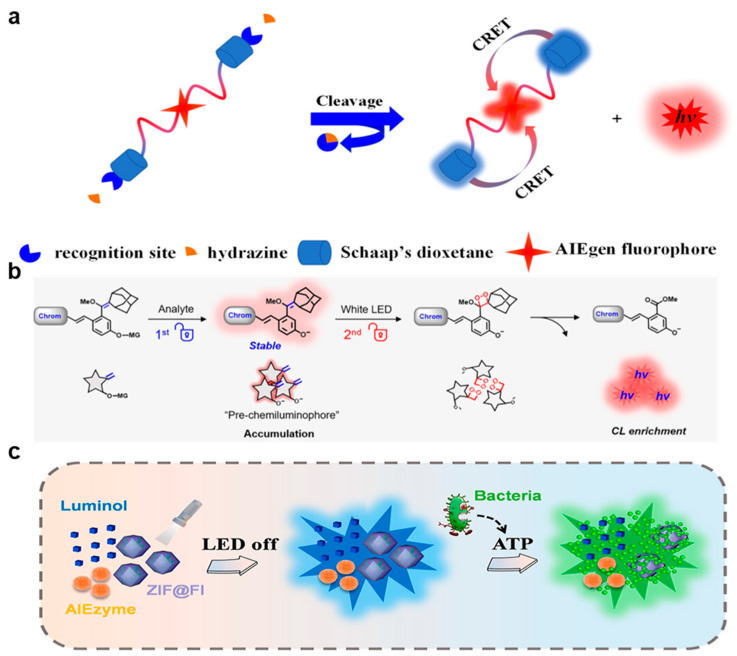
(**a**) Reaction process of the photoactivatable chemiluminescent AIEgen probe for hydrazine detection, from [7]. (**b**) The dual-lock strategy: first, the masking group is triggered and removed by the analyte, leading to the generation and accumulation of stable pre-chemiluminophores. Second, pre-chemiluminophores are triggered by LED light irradiation for the generation of high-energy 1,2-dioxetane, leading to the enrichment of CL signals, from [8]. (**c**) Schematic illustration of the ATP-triggered bacterial detection nanoplatform based on the CRET principle, from [9].

The sensitive detection of hydrazine is of significant importance, as when it is absorbed by the skin, respiratory system, or digestive system, serious damage can be caused to the human organs and nervous system [57]. The United States Environmental Protection Agency has listed hydrazine as a potential carcinogen with a threshold of 10 ppb [58]. Therefore, it is very meaningful to develop a simple, specific, and sensitive method for the detection of hydrazine in environmental and biological systems. Li’s group developed a new type of light-activated red CL-AIE probe (ACL) (as shown in Figure 4a) [7]. The C=C bond connected to adamantane in the ACL probe was converted to dioxane by ^1^O_2_ generated from the AIE photosensitizer after irradiation to form an activated CL-AIE probe (ACLD). In the presence of hydrazine, the acylated phenolic hydroxyl group in the ACLD probe was removed, leading to a self-immolation reaction, which can release high energy for the chemical excitation of 1,2-dioxetanes. A red-colored CL signal was then emitted through the transfer of intramolecular CRET from Schaap’s dioxetane to the red-color-emissive AIE photosensitizer. Furthermore, the ACLD can detect hydrazine with high specificity because its ultrahigh CL signal can be triggered in the presence of 200 µM hydrazine, while other analytes (such as aniline, urea, hydroxylamine, ethanolamine, triethylamine, F^−^, Cl^−^, Br^−^, I^−^, CH_3_COO^−^, CO_3_^2−^, NO_3_^−^, SO_3_^2−^, SO_4_^2−^, S^2−^, HS^−^, Li^+^, Na^+^, K^+^, NH_4_^+^, Ca^2+^, Mg^2+^, Cu^2+^, Fe^2+^, Fe^3+^, Al^3+^, Cys, GSH, Gly, Ile, Pro, Phe, Ser, and Lys) can not. The CL intensity of the ACLD solution did not decrease significantly during the storage time of 14 days, indicating its excellent stability and photoactivity. The sensitive detection of hydrazine was realized in vitro and in vivo, with a detection limit down to 0.18 µM (5.72 ppb), lower than the standard of the United States Environmental Protection Agency and that of the previously reported fluorescence method [59].

CL-based technologies have revolutionized the monitoring of biomolecules in vitro. However, significant technical hurdles have limited the achievement of trigger-controlled, bright, and enriched CL signals and the instability of high-energy units in chemiluminescence reactions makes it difficult to synthesize chemiluminescence probes with the desired stability. Guo et al. designed a novel dual-lock photoactivable CL probe (Figure 4b) [8]. In this dual-lock strategy, the retention ratio increased from 5% to 91% after continuous illumination for 60 min, showing that a C=C double bond instead of the dioxetane group for DCM-gal-CF can display much higher stability. In the presence of analytes such as galactosidase, the galactose moiety in the probe was specifically enzymatically hydrolyzed and removed, resulting in the formation and accumulation of pre-CL fluorophore with an AIE property. Then, the oxidation of the electron-rich double bonds by ^1^O_2_ to produce 1,2-dioxane in situ led to a strong CL signal after the detaching of Schaap’s dioxetane. Dual-mode bioimaging combining real-time AIE fluorescence and an ultra-sensitive CL signal was realized in the in vivo tumor tracking.

### 3.4. Pathogen Assay

Pathogens are widely distributed in different environments, which can bring serious threats to human life and health [47,60]. Point-of-care testing (POCT) is a valuable method for early warning of bacterial threat. Li’s group constructed a CL-based ratiometric sensing platform for bacteria detection based on an AIE enzyme (called AIEzyme) that exhibited oxidase-like properties (as shown in Figure 4c) [9]. It could emit luminol CL that was visible to the naked eye for 2 h in the absence of hydrogen peroxide. This phenomenon can be attributed to the persistent ROS produced by the cyclic energy transfer between AIEzyme and luminol. The platform was successfully applied for sensitive bacterial POCT, with a detection limit down to 1.74 CFU·mL^−1^ in the testing of *E. coli* in tap water and urine.

## 4. In Vivo Bioimaging and Image-Guided Therapy

### 4.1. External Light-Triggered CL Imaging and Disease Therapy

Efficient NIR afterglow luminescent materials and probes have great prospects. Their applications in biomedical fields, such as accurate image-guided cancer surgery, are being pursued by scientists. Ding’s group designed and synthesized novel afterglow luminescent nanoparticles (namely AGL-AIE nanodots) by encapsulation of an NIR AIE photosensitizer and the CL substrate Schaap’s dioxetane within Lipid-PEG_2000_ surfactants, which was also verified to possess excellent photostability as evidenced by the negligible change in hydrodynamic diameter within 7 days [15]. After a short period of external light irradiation, the AGL-AIE nanodots could emit an NIR afterglow luminescence lasting more than 10 days through a series of processes including singlet oxygen production by AIE photosensitizer, formation of Schaap’s dioxane, chemical excitation of dioxane decomposition, and energy transfer back to the NIR AIEgens. TPE-DCM has a great capability for the generation of ^1^O_2_, which is stronger than the commercial photosensitizer Rose Bengal. Because of its ultrahigh tumor-to-liver signal ratio and high signal-to-noise ratio, AGL-AIE nanodots showed excellent performance in accurate image-guided cancer surgery.

**Figure 5:**
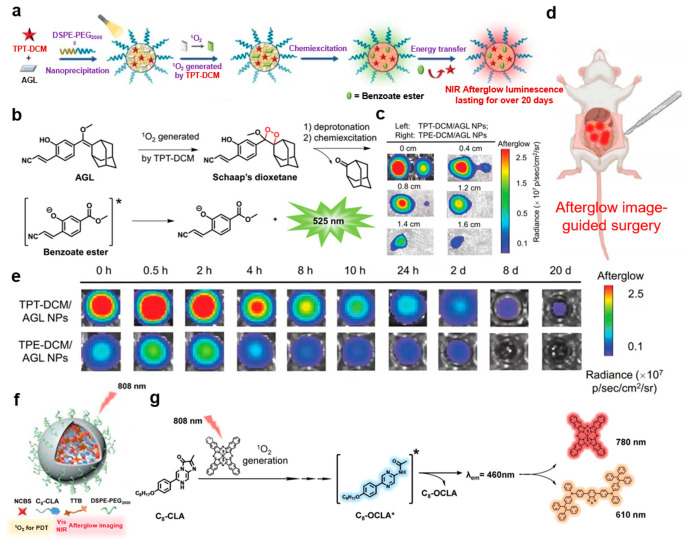
(**a**) Schematic illustration of the formation of TPT-DCM/AGL NPs and generation of afterglow luminescence. (**b**) Chemiexcitation mechanism of compound AGL. (**c**) Afterglow images of TPT-DCM/AGL NPs (**left**) and TPE-DCM/AGL NPs (**right**) covered by chicken tissue with different thicknesses in PBS at 37 °C post white light pre-irradiation (0.2 W·cm^−2^, 2 min). (**d**) Schematic illustration of afterglow luminescence image-guided surgery. (**e**) Partial time-dependent afterglow images of TPT-DCM/AGL NPs and TPE-DCM/AGL NPs in PBS at 37 °C post white light pre-irradiation (0.2 W cm^−2^, 2 min). (**a**–**e**) were modified from [16]. (**f**) Synthesis of the afterglow organic nanoparticles (ONPs) for afterglow imaging-guided PDT. (**g**) Detailed mechanism of the afterglow luminescence generated by the ONPs. * means excited state of C_8_-OCLA. (**f**,**g**) were modified from [14].

In order to improve the quality of image-guided cancer surgery, it is particularly important to develop luminescent materials with longer emission life and deeper tissue penetration capability. As shown in Figure 5a, Liu’s group reported a triazole-based NIR AIEgen (TPT-DCM) with high molar extinction coefficient, strong brightness, and efficient ROS generation capability [16]. These characteristics enable it to function as a nanoprobe with an NIR afterglow luminescence up to 20 days and a tumor-to-liver signal ratio up to 187 (Figure 5e). It was used for afterglow imaging of tissues up to 1.6 cm thickness and long-term imaging via CRET initiated by the active Schaap’s dioxetane (Figure 5b,c). In addition, TPT-DCM/AGL nanoparticles had good colloidal stability, as the hydrodynamic diameter and Zeta potential remained unchanged for 7 days. Due to these excellent performances, surgical navigation guided by afterglow imaging was able to successfully remove tumors (Figure 5d).

Optical imaging-guided photodynamic therapy (PDT) is a new technology for tumor therapy, which has the advantages of accurate tumor targeting and non-invasive therapy. However, due to the low signal-to-background ratio caused by the auto-fluorescence in biological tissues, most luminescence imaging systems showed low sensitivity in imaging deep tumor tissues in vivo. Tong’s group synthesized organic nanoparticles (ONPs) with persistent NIR emission with a half-life of several minutes (shown in Figure 5f,g) [14]. They were used in afterglow imaging to guide the photodynamic therapy of a xenograft HeLa tumor mouse model. ONP in vivo afterglow tumor imaging has the advantages of high SBR, good tissue penetration, and robust singlet oxygen generation efficiency, realizing excellent performance in inhibiting tumor growth in mice with minimal damage to major organs.

### 4.2. CL Imaging and Disease Therapy without External Light Triggering

**Figure 6:**
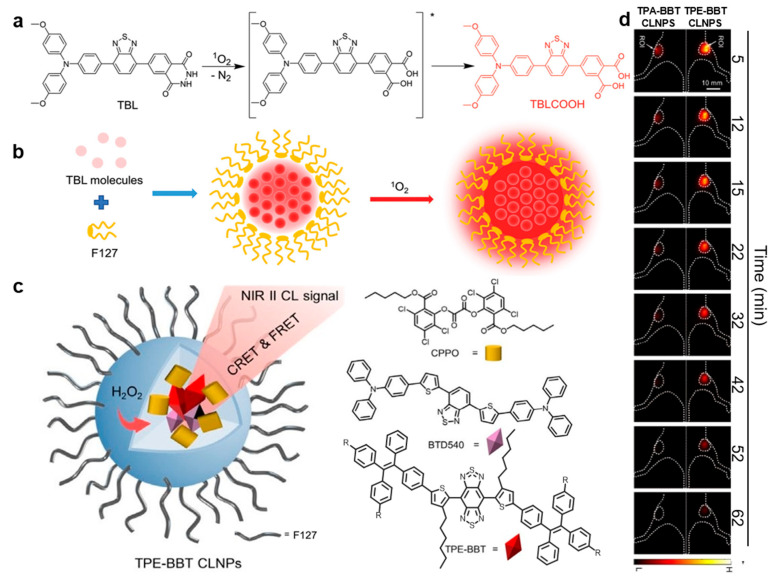
(**a**) The proposed CL generation mechanism of TBL oxidized by ^1^O_2_. * means excited state of TBL. (**b**) Schematic illustration of the preparation of TBL dots and the generation of CL. (**a**,**b**) were from [10]. (**c**) Schematic illustration of the fabrication of TPE-BBT CL nanoparticles (CLNPs) using F127 as the surfactant. (**d**) In vivo NIR-II CL imaging of arthrosis inflammation using TPE-BBT and TPA-BBT CLNPs at different postinjection times. Exposure time: 10 s. (**c**,**d**) were modified from [11].

NIR CL emission is very suitable for deep tissue imaging as no external light is needed for its excitation and due to the low scattering of NIR light in biological tissues [61]. Tang and Liu designed a NIR CL unit, TBL, with AIE activity (Figure 6a) [10]. In the TBL molecule, the luminol unit was coupled with the electron receptor benzothiadiazole and electron donor triphenylamine. Using F127 as a surfactant, the TBL nanodot was prepared via co-precipitation in the mixture of water and THF under ultrasonic conditions (Figure 6b), and no precipitation was observed when the nanoparticles were stored in PBS and DMEM solutions for 4 weeks, indicating the excellent stability of the nanoparticles. The CL of TBL nanodots can last for more than 60 min, and its NIR CL can penetrate tissues with a total thickness of more than 3 cm. It can be used in in vitro and in vivo detection of ^1^O_2_. In addition, the body weight of tumor-bearing mice and healthy mice did not change significantly for 7 days after injection of TBL dots, indicating the excellent biocompatibility of TBL dots. In particular, in vivo CL imaging can successfully distinguish tumors from normal tissues, suggesting great potential in the application of CL-guided tumor diagnosis and surgery.

The design of fluorophores in the second NIR (NIR-II) window with high quantum yield has a good prospect in clinical application. Tang and Shen replaced the TPA group in TPA-BBT with a TPE group to synthesize two NIR-II emitters, called TPE-BBT and TPEO-BBT (Figure 6c) [11]. The QY of the photoluminescence of the prepared nanoparticles (d < 30 nm) from TPE-BBT and TPEO-BBT with the help of the F127 surfactant (PLNPs) were 31.5% and 23.9%, respectively, much higher than that of the TPA-BBT PLNPs and commercial IR26 (QY = 0.5%). In addition, the absolute QY of the TPE-BBT crystal was 10.4%, which is the highest absolute QY among the reported NIR-II systems. Compared to TPA-BBT and commercial ICG, TPE-BBT showed better performance with much higher SBR and imaging quality (Figure 6d), indicating its potential in the field of bioimaging. Furthermore, the signal of commercial NIR contrast agent indocyanine green (ICG) decreased significantly after 30 min irradiation, while the signals of TPE-BBT and TPEO-BBT PLNPs almost did not change, indicating the better photostability of the AIE systems. Moreover, the SBR of the CL signal was still greater than 10 at 62 min after injection, indicating that it has a long-term CL and good imaging quality.

Because CL imaging is not interfered with by autofluorescence as no excitation source is needed in its excitation, this leads to a significantly improved signal-to-noise ratio. However, due to the strong scattering and absorption of tissue, the CL imaging of visible light and the first NIR-I region often can not realize the desired imaging results in deep tissues. Wang’s group designed a self-luminescent NIR-II CL nanoprobe that can emit NIR-II luminescence in the presence of hydrogen peroxide [12]. During the seven-day storage of the NIR-II CL nanoprobe, the Zeta potential drift was negligible, indicating the excellent colloidal stability of the NIR-II CL nanoprobes. Six batches of NIR-II CL nanoprobes synthesized by the same experimental procedure showed chemiluminescence signals of similar intensity, with a relative standard deviation of 4.2%, indicating that they possessed excellent reproducibility. Furthermore, within the testing concentration range of the nanoprobes, the survival rates of human embryonic kidney cells in the experimental group were more than 85%, indicating their good biocompatibility and low cytotoxicity. The CRET from the CL substrate to the NIR-I organic molecule and Förster resonance energy transfer (FRET) from the NIR-I organic molecule to the NIR-II organic molecule sequentially took place in the nanoprobe by emitting a bright NIR-II fluorescence signal for the deep tissue imaging. Based on their high selectivity and sensitivity to hydrogen peroxide (the detection limit was as low as 44.4 nm) and their long-term luminescence properties, the prepared NIR-II CL nanoprobes were successfully applied in the detection of inflammation in mice. Compared to the single fluorescence mode, the signal-to-noise ratio of this NIR-II CL system was increased by 7.4-fold, indicating its great potential in in vivo applications.

Alternatively, Zhang’s group proposed a new CL sensor, which works in the NIR-II range and has a capability for penetration to deep tissue depth (8 mm) [13]. Successive CRET and FRET occurred among CPPO and two delicately designed donor–acceptor–donor fluorophores, BTD540 and BBTD700. The sensor could selectively detect the H_2_O_2_ in the local inflammation of mice, whose signal-to-noise ratio was 4.5-fold higher than that of the single NIR-II fluorescence mode. In addition, there is no obvious change in the size of NIR-II CLS after storage in water for 10 days, indicating the good colloidal stability of NIR-II CLS systems.

**Figure 7:**
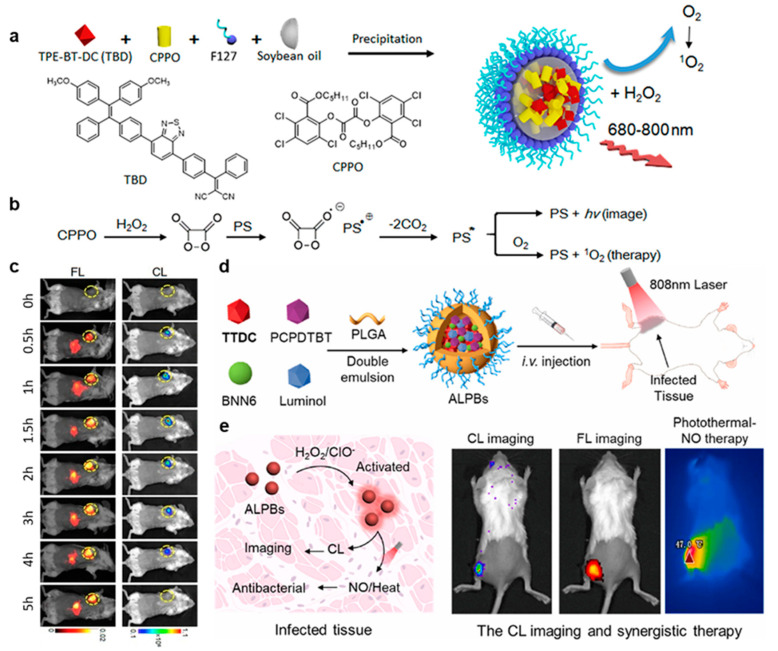
(**a**) The preparation of C-TBD NPs. (**b**) Illustration of the principle for CL and ^1^O_2_ generation of C-TBD NPs in the presence of H_2_O_2_. (**c**) Time-dependent in vivo FL and CL imaging of mice receiving C-TBD NPs (1 mg·mL^−1^ based on C-TBD, 100 μL per mouse) over 5 h periods. Tumor regions are marked with yellow circles. PS* means excited state of PS. (**a**–**c**) were modified from [17]. (**d**) ALPBs were synthesized by a double-emulsion method using PLGA-PEG5000 as a matrix to encapsulate TTDC, luminol, BNN6, and PCPDTBT, which was applied to CL imaging and synergistic photothermal–NO therapy of bacterial infection. (**e**) Once intravenously injected into the bacteria-infected mouse body, ALPBs were activated by elevated ROS to generate tissue-penetrating NIR CL through the CRET process. Under the guidance of imaging, synergistic photothermal–NO therapy could be conducted by 808 nm laser irradiation. (**d**,**e**) were from [18].

Due to the complexity of designing long-wavelength CL systems and the potential biological toxicity of some chemical reactions during the excitation of the CL substrate, probes for CL-guided photodynamic therapy in vivo require careful design. Liu’s group reported a novel type of CL nanomaterial with chemical reaction-excited far-red/NIR CL emission and ^1^O_2_ production [17]. The ^1^O_2_ generation efficiency of C-TBD NPs is 2.7-fold higher than that of Ce6, which is one of the most frequently used PSs. CPPO and the AIE photosensitizer TBD were co-encapsulated with surfactants Pluronic F-127 and soybean oil to form C-TBD nanoparticles (Figure 7a). In addition, there was no weight loss and no significant damage to the main metabolic organs in normal mice treated with C-TBD, indicating its good biocompatibility and low biotoxicity. The presence of soybean oil largely enhanced the half-life from 1 h for TBD NPs to 2.3 h for C-TBD NPs. In the presence of hydrogen peroxide, C-TBD nanoparticles can emit red/NIR CL signals and produce a large amount of ^1^O_2_, which can be used for accurately tracking tumors in vivo and at the same time inducing tumor cell apoptosis and inhibiting tumor growth, providing a new strategy for intelligent, accurate, and non-invasive tumor therapy (Figure 7b,c).

Bacterial infection is the leading cause of many inflammatory diseases that seriously threaten human health [62]. Existing methods for the treatment of bacterial infections are always complex and suffer from compromised antibacterial efficiency [63]. As shown in Figure 7d, Liu’s group reported NIR CL nanoparticles, denoted as ALPB, containing a CL substrate (luminol), an AIE-active fluorophore (TTDC), an NIR-emissive and photothermal-active agent (PCPDTBT), and a heat-responsive nitric oxide (NO) donor (BNN6) [18]. The cascade energy transfer between luminol and PCPDTBT was bridged by TTDC as the overlap of its absorption and emission spectra with the luminol’s emission and PCPDTBT’s absorption, respectively (Figure 7e). The nanoparticles accumulated at the infected site after intravenous injection and were then activated by the oversecreted ROS in situ to produce NIR CL. ALPBs were proven to be capable of accurately tracking the local inflammation induced by infection. Image-guided photothermal–NO gas therapy under 808 nm laser irradiation can effectively remove the bacteria and quickly recover the infected tissue. On the other hand, incubation with ALPs and ALPBs had little effect on the activity of *S. aureus* in solution, indicating its excellent biocompatibility.

## 5. Conclusions and Prospects

In summary, following the above developments of AIE-based CL systems and their distinctive advantages summarized in Table 1, the limitations and future prospects are also discussed. The examples of AIE-based CL systems are still very limited, and we expect they are worthy of further study and exploration in terms of the following aspects. First of all, the structures and properties of AIE-CL systems are as follows:

(1) The range of substrates reported in AIE-CL systems is still limited to several common CL molecules, such as luminol, peroxalic acid, dioxetanes, etc., and the response of triggering CL is also limited to a very small amount of active substances, such as hydrogen peroxide and singlet oxygen. We believe that expanding the substrate range of AIE-CL systems and exploring more response modes is of great significance in the development of new CL systems with expanded applications.
(2) It is well known that in order to facilitate deep tissue imaging, the development of NIR luminescence imaging dyes has always been pursued by researchers. However, the emission wavelengths of existing AIE-CL systems are generally located in the visible range. Although there are a few reports of CL with long wavelength emission, they still need to transfer the CL wavelength to the NIR range with the help of CRET/FRET, which is seriously limited by several critical parameters, including the number ratio of donors and acceptors, spatial distance, overlap coefficient of the emission spectra of donor and the absorption spectra of the acceptor, etc., and often requires complex and tedious molecular or nanostructural design. Therefore, the design of single molecular AIE-CL imaging substrates with NIR emissions is of particular importance and has application potential.
(3) Generally speaking, CL systems can produce light signals without external light excitation, but at the same time, CL signals often have poor capability to resist environmental interferences. Therefore, it is promising to design novel CL dyes with stabilized CL signals. The strategy of combining AIE systems with CL to make aggregated-state CL molecules has been proven to be feasible for enhancing the anti-interference ability of CL molecules to some extent. In addition, grafting CL molecules onto classical luminescent agents with verified stability (e.g., nanocluster, quantum dots) is likely to increase their photo- and structural stability.

On the other hand, the following must be taken into account in the function and application of AIE-CL systems:

(1) In the previous reports, most of the developed AIE-CL systems were responsive to a single active substrate. Provided they can respond to more diverse targets or to multiple targets at the same time, they will become a more powerful tool in the field of biochemical analysis and bioimaging and detection;
(2) The combination of multimodality signals can make use of the complementary effects of each individual imaging signal with different characteristics, resulting in significantly improved sensitivity, accuracy, and specificity in bioimaging. Therefore, the construction of a multimodality bioimaging platform, such as combining the highly sensitive CL signals of an AIE-CL system with magnetic resonance imaging (MRI) and computed tomography (CT) signals with the characteristics of high penetration depth and high spatial resolution can provide more effective candidates for accurate bioimaging. At the same time, the signal generated by a CL system without external light excitation is converted into a photothermal effect in situ, leading to the generation of an ultrasonic signal with the capability of deep-depth tissue penetration. Thus, it can realize combined CL and PA signals multimodal bioimaging;
(3) Most of the developed AIE-CL systems only have an imaging function, but there are few reports of AIE-CL systems with therapeutic functions. The further incorporation of therapeutic functions into the existing AIE-CL imaging systems, such as PDT and RT, can not only have excellent imaging effects on disease tissues, but also achieve image-guided disease treatment, holding great potential to provide new strategies for applications in disease detection and treatment.

## Figures and Tables

**Table 1:** The advantages of individual AIE-based CL systems.

	Advantages	Reference
1	Fast, sensitive, and selective detection of Hg^2+^; a linear detection range of 0.005–10 μg mL^−1^ and limit of detection (LOD) of 3 ng mL^−1^	[30]
2	Detection range of 2.5–125 μg/L CN^−^ with a LOD down to 0.55 μg/L; high sensitivity, high selectivity, and anti-interference capability	[31]
3	Detection range of NaNO_2_ from 1.0 to 100 μM with a LOD as low as 0.5 μM; recovery was as high as 98−106% and accuracy was good	[36]
4	Remarkably enhanced CL signals and faster reaction rate	[4]
5	Low cytotoxicity and good animal compatibility; high energy transfer efficiency; high CL amplification; LOD was as low as 4.6 × 10^−9^ M	[5]
6	Highly sensitive to O_2_^•−^ with LOD of 0.21 nM for FL and 0.38 nM for CL	[6]
7	Simplicity, good specificity, and sensitivity for the detection of hydrazine; good stability and photoactivity; LOD down to 0.18 µM (5.72 ppb)	[7]
8	Stimuli-controlled, bright, and enriched CL signals with advantages in stability, brightness, and imaging flexibility	[8]
9	Long persistent luminescence; strong CL intensity; excellent capability of ROS generation; good anti-interference capability; outstanding stability; free of H_2_O_2_ and external light sources; high detection accuracy	[9]
10	High NIR afterglow luminescence persisting over 10 days; deeper tissue penetration; ultrahigh tumor-to-liver signal ratio; low afterglow background noise	[15]
11	High molar extinction coefficient; good brightness; excellent reactive oxygen species generation rate; ultralong NIR afterglow luminescence (up to 20 days); ultrahigh tumor-to-liver signal ratio (up to 187-fold)	[16]
12	Persistent luminescence; good biocompatibility; high SBR; good tissue penetration; abundant singlet oxygen generation	[14]
13	High NIR CL emission; tissue penetration depth of over 3 cm	[10]
14	Ultrahigh relative QYs; high signal-to-background ratio; high energy transfer efficiency; excellent continuous imaging	[11]
15	Excellent selectivity; high sensitivity to hydrogen peroxide; long-lasting luminescence performance; high signal-to-background ratio	[12]
16	Deep penetration depth; high signal-to-background ratio; large Stokes shift (>100 nm) and extremely high FRET efficiency (94.12%)	[13]
17	Bright FR/NIR self-luminescence and significant ^1^O_2_ production in the presence of H_2_O_2_	[17]
18	Excellent photothermal conversion efficiency; simultaneous CL and photothermal–NO therapy for deep tissue infection	[18]
